# Combining social cues in attention: Looking at gaze, head, and pointing cues

**DOI:** 10.3758/s13414-023-02669-6

**Published:** 2023-02-27

**Authors:** Zhifan Lu, Wieske van Zoest

**Affiliations:** School of Psychology, University of Birmingham, Edgbaston, Birmingham, B15 2TT UK

**Keywords:** Social attention, Central cuing paradigm, Gaze cue, Head cue, Pointing, Competing cues

## Abstract

Social cues bias covert spatial attention. In most previous work the impact of different social cues, such as the gaze, head, and pointing cue, has been investigated using separated cues or making one cue explicitly task relevant in response-interference tasks. In the present study we created a novel cartoon figure in which unpredictive gaze and head and pointing cues could be combined to study their impact on spatial attention. In Experiment 1, gaze and pointing cues were either presented alone or together. When both cues were present, they were always directed to the same location. In Experiment 2, gaze and pointing cues were either directed to the same location (aligned) or directed to different locations (conflicted). Experiment 3 was like Experiment 2, except that the pointing cue was tested alongside a head-direction cue. The results of Experiment 1 showed that the effect of the gaze cue was reliably smaller than the pointing cue, and an aligned gaze cue did not have an additive benefit for performance. In Experiments 2 and 3, performance was determined by the pointing cue, regardless of where they eyes were looking, or the head was directed. The present results demonstrated a strong dominance of the pointing cue over the other cues. The child-friendly stimuli present a versatile way to study the impact of the combination of social cues, which may further benefit developmental research in social attention, and research in populations whose members might have atypical social attention.

**Supplementary Information:**

The online version contains supplementary material available at 10.3758/s13414-023-02669-6.

Recognizing social cues in the environment facilitates efficient interactions with others in everyday life. Social cues, such as gaze, head, and pointing, convey important directional information to potential shared regions of interest. Evidence suggests that the combination of two cues may facilitate attentional performance when they are directed to the same location. In conflicting situations where two cues are directed to different location, the attention system may prioritize the processing of one cue over the other (e.g., Langton & Bruce, 2000; Langton et al., 2000). Results have demonstrated that social cues have a considerable impact on reaction times in visual detection or discrimination tasks, showing that valid cues lead to faster responses than invalid cues (e.g., Driver et al., 1999; Friesen et al., 2005; Gregory et al., 2015). However, most studies have explored the role of different social cues in isolation, using disjoined stimuli (e.g., Friesen et al., 2005; Friesen & Kingstone, 1998; Hermens et al., 2017; Langton & Bruce, 1999; Sato et al., 2007) or made cues explicitly task relevant for response selection (e.g., Langton & Bruce, 2000). The aim of the current study was to integrate multiple social cues and evaluate their combined influences on biases of spatial attention.

Gaze cues can lead to reflexive covert attentional shifts, even when they are uninformative and participants are instructed to ignore the directional information (e.g., Friesen & Kingstone, 1998; Ristic et al., 2007; Tipples, 2005). The spatial cueing paradigm is often used to investigate shifts of attention probed by central cues. For example, in an experiment by Friesen et al. (2005), a schematic face appeared in the centre of the screen. After a brief period in which the gaze was neutral, the pupils in the eyes would shift to depict a look to the left or right side of space. Participants were instructed to respond to a specific target, which would appear either at a location looked at by the gaze cue (validly cued location) or the opposite location (invalidly cued location). The results showed that even when respondents were informed that the cues were unpredictable and had no informative value regarding the upcoming target location, targets shown at cued locations elicited faster response times than targets provided at uncued locations.

A pointing hand or finger has similarly been shown to act as an important social cue and automatically modulate covert attention (Ariga & Watanabe, 2009; Gregory & Hodgson, 2012; Sato et al., 2010; Tomonaga & Imura, 2009). Sato et al. (2010) used a central cueing paradigm, in which participants were presented with images of a hand with an index finger, asking them to recognize target items that randomly appeared around the cue. Comparing validly cued to invalidly cued target, the results demonstrated a robust orientation effect of pointing cues. In young children, the influence of pointing cue of overt eye-movement selection has been demonstrated as consistently stronger than gaze cues (Gregory et al., 2016). Studies using head direction as central cue have revealed comparable benefits for spatial attention when this cue correctly indicates the upcoming location of a target (Cooney et al., 2017; Langton, 2000; Langton & Bruce, 1999). Langton and Bruce (1999) showed participants a face in the centre of the screen either looking upwards, downwards, to the left, or to the right. Participants were asked to press the space bar on a keyboard as soon as they detected a target letter which could appear at one of four locations. While the cue was uninformative regarding the upcoming target location, the results showed that participants were faster to detect the target when it appeared at a cued location, especially when the target was presented shortly after the presentation of the cue. The authors furthermore showed that head direction cannot be ignored when presented as an irrelevant distractor (Langton & Bruce, 2000). In this experiment, participants were presented with photos of an individual who was orienting their head either upwards or downwards, while superimposed on the photo a white arrow was presented that could be congruent or incongruent with the direction of the head. While observers were instructed to ignore the head cue and instead respond to the direction of the arrow, the results indicated that the irrelevant head cues interrupted participants’ performance to the arrow cues. Note, this latter experiment did not test the impact of the cue on spatial attention, but rather how quickly observers responded to the direction of the cue.

There is evidence to suggests that when two different cues are aligned in direction and both cue the same location, the resulting impact on response selection might outweigh responses given to a single cue in isolation. Langton and Bruce (2000) exhibited photos of individual whose head and hands were simultaneously pointing in the same (congruent) or opposite (incongruent) location. They discovered when the cues were consistent, the directional response was greatly facilitated compared with when one of cues was neutral or incongruent. While this suggests a benefit for two cues compared with one when they are congruently aligned to the same location, there exists disagreement over how these cues are processed when they occur in competitive situations. Some have argued that cues, such as head orientation, gaze direction, directional gestures, and spoken language, are processed independently by separate mechanisms (Carlin et al., 2012; Langton & Bruce, 1999; Materna et al., 2008). Consequently, when multiple conflicting cues are present simultaneously, the attentional system may prioritize allocating resources to a one specific cue. In an experiment by Langton and Bruce (2000), participants were told to respond either to the head direction or pointing direction of a central image. In the images that were presented, the person was pointing either upwards or downwards whilst orienting their head in the same direction as their pointing gesture or in the opposite direction to their pointing gesture. The results showed that when observers were responding to head direction incongruent pointing cues interfered much more strongly with response-selection compared with when observers were responding to the pointing direction and the head cue was incongruent. This result suggests that pointing gestures create stronger response biases than do cues elicited by head orientation.

In contrast to studies which focussed mostly on response selection, Hermens et al. (2017) presented head and gaze cues either both pointing to the same location or competing direction to investigate how spatial attention is affected by multiple cues. Unlike Langton and Bruce (2000), in their experiment they used separate images to depict the eyes, hand, face and head cues. They found that head cues significantly interfered with subjects’ responses to gaze cues and did so to a greater degree than gaze cues did with head cues. This work suggests that the head cue may be a more powerful cue than the gaze cue when both are presented together. Hermens et al. (2017) suggest that the impact of cues may depend on whether they have a clearly defined outline relative to the background. In this sense, the head cue, but also pointing and arrow cue, have distinct outlines, whereas the gaze cue does not have a distinct outline as the cue is integrated in the face. Their results suggest that cues with a distinct outline are more competitive in conflicting situations than cues without a distinct outline. Then again, recently Kajopoulos et al. (2021) evaluated the influence of distracting gestures (pointing finger) on attentional focus during realistic interactions and observed that the willingness to attend to an interacting partner’s gaze was not disrupted by the inconsistent pointing gesture.

In sum, the literature reveals incongruities regarding the impact of multiple social cues on spatial attention. This may be the result of methodological differences between studies. In some studies, social cues were presented as separate images (e.g., Gregory & Hodgson, 2012; Hermens et al., 2017; Sato et al., 2010). Other studies used integrated real-life photos, but here one of social cues was made explicitly task relevant while the other cue was presented as an irrelevant distractor (e.g., Langton & Bruce, 2000). This latter task is more akin to a response-selection interference task (e.g., like the Stroop task; for a review see MacLeod, 1991), compared with a spatial cueing task which measures the consequence of the cue on visual spatial attention. While previous work provides important information regarding the ability to strategically use social cues, and ignore them when they are potentially distracting, it is unclear how social cues jointly affect biases in spatial attention.

The aim of the present study was to study the combined effects of facial and gestural cues via a central cueing paradigm, where cues were unpredictive and completely irrelevant to the task. A novel cartoon character was developed in which gaze-direction and pointing (Experiments 1 and 2) and pointing and head-direction were manipulated (Experiment 3). In Experiment 1, cues were either presented individually or were presented together to test whether the effect of the two cues are additive, or whether one cue is dominant in driving performance. In Experiment 2, two cues were presented, but they could be aligned and directed at the same location or conflicted and directed at two different locations. Like Experiment 1, this experiment was aimed to provide further insight into the potential differences in priority in the processing of the gaze and pointing cue. Experiment 3 was similar to Experiment 2, but this time a head-direction cue was posed against the pointing cue.

## Experiment 1

In Experiment 1, we employed the cueing paradigm to examine how unpredictable (1) gaze cues, (2) pointing cues, and (3) the combination cues (in which the gaze and hand cue were presented concurrently and directed to the same location) affect spatial attention. In addition to the three conditions, a neutral cue was presented which contained no directional information. It was predicted that if two cues directed to same location are better than one cue, an overall benefit should be found for the combination cue compared with the cues presented in isolation.

Moreover, in Experiment 1, target and distractor were presented at four stimulus positions (upper left, upper right, lower left, lower right). This manipulation was motivated by the idea that there are processing benefits for the upper visual field in visual search (e.g., Previc, 1990; Thomas & Elias, 2011) and allowed us to explore the effect of visual fields on gaze and pointing cues.

### Methods

#### Participants

The SONA platform recruited 32 students (23 female; mean age 19 years) in exchange for course credit. Each subject had normal or corrected-to-normal eyesight. Participants used their personal computer (PC) equipped with a keyboard to complete this experiment. Informed consent of each participant was obtained prior to the study; the study was approved by the university Ethical Committee (protocol ERN-19-0260) and was conducted in accordance with the principles expressed in the Declaration of Helsinki.

Sample size was predicted based on previous study that combined gaze and arrow cues, looking for a similar combination effect (Nummenmaa & Hietanen, 2009, Experiment 1). In this work the primary effects of cue validity had an effect size of η^2^ = 0.69. To obtain the anticipated statistical power of 0.9 (Anderson et al., 2017), the current investigation required minimum sample size of 16 participants (alpha = 0.01). As a result, the current sample size of 32 patients was judged sufficient to evaluate the effect.

#### Apparatus

This online experiment was conducted utilizing the platforms OpenSesame and OS Web (Mathôt et al., 2012), JATOS (Lange et al., 2015), and Qualtrics (qualtrics.com) on the personal computers of participants.

#### Stimuli

**Fig. 1:**
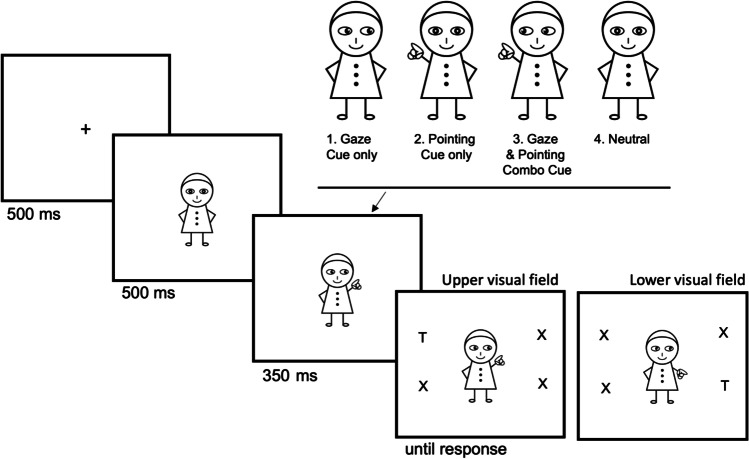
The trial sequence in Experiment 1. Four types of cue condition were presented: (1) Gaze Cue only, (2) Pointing Cue only, (3) Gaze & Pointing Combo Cue, and (4) Neutral cue. Targets and distractors appeared in the upper or lower visual field on either the left or right side of space. Cue validity was 100% valid with respect to the upper/ lower field, but 50% valid with respect to the left/ right side of space. Note, stimuli are not drawn to scale

In this experiment, social cues were manipulated using a cartoon character which conveyed directional information via changes in gaze and pointing gestures. There were four cue conditions: gaze-only cues, pointing-only cues, combination cues, and a neutral condition (Fig. 1). Both the pupils of the eyes and arms were manipulated to indicate a cue in either the upper or lower visual field. The vertical movement angle of the eyeballs, arms, and hands would be 45° or 135°, indicating direction either to the upper or lower visual field. The target could appear at four different locations: upper left, lower left, upper right, and lower right. Note that the hands and eyes in the combined cue were always oriented in the same angular direction. The cues were 100% valid with respect to the hemifield—that is, directed validly to the upper or lower field. The cues were 50% with respect to the left or right side of space.

All cartoon graphics were presented on a white background, and the figures, target, and distractor letters were all black. The target letter, an uppercase “F” or “H” was assigned to one of four alternative places with equal frequency across trials (Fig. 1). Three distractor letters, “X”, were placed alongside the target letter in the remaining three locations.

#### Design

Experiment 1 contained two within-subject factors: cue type (gaze only, pointing only, or combo cue) and validity (valid or invalid). In half of the trials, the target letter appeared on the side to which the cue was directed (validly cued trials), but in the remaining trials, it appeared in the opposite position (invalidly cued trials). In other words, the cues were completely unpredictive (50% of overall trial count) of the side of the screen of upcoming target. The target was 100% valid with respect to the upper or lower visual field. That is, in response to an “upper-oriented” cue, the target would appear in either the upper-left or upper-right location, but never in the lower field. The two target letters were located an equal number of times at the upper or lower of the visual field. In 25% of the trials (i.e., 64 trials) a neutral stimulus was presented containing no directional information, which served as a baseline condition.

#### Procedure

Participants supplied informed consent prior to the start of this online experiment. Participants were instructed to sit in front of a table equipped with a computer at a distance approximately 60 cm from the screen. The participants were asked to fixate the central fixation point throughout the experiment, then maintain fixation, and respond as quickly and precisely as possible when the target stimulus occurred. Additionally, they were told that these social cues were uninformative and that they should ignore them.

Each trial was initiated by the appearance of a fixation point in the centre of the screen (Fig. 1). Five hundred milliseconds later, the neutral cartoon figure was presented for 500 ms which contained no directional information. Subsequently, one of the different types of cues was displayed randomly—gazing, pointing, the combo cue, or neutral—for 350 ms, following which the target letter (“F” or “H”) appeared in one of four possible positions surrounding the central cue. The other three locations contained nontargets (letter “X”). Once the target appeared, participants were instructed to identify the letter and indicate whether it was an “F” or an “H” by pressing the corresponding key on the keyboard (“f” or “h”). Each participant would first complete 16 practical trials, followed by 256 experimental trials divided into 16 blocks. Cue type and validity were randomly mixed within the blocks of trials. Participants received feedback on their average reaction time and accuracy following each block and were then free to relax.

#### Analyses of data

The reaction time (ms) and accuracy were collected using the OS Web (OpenSesame) and JATOS software. MATLAB was used to extract and clean up the gathered data. JASP software (JASP Tea, 2022) was used to conduct statistical analyses.

### Results

Four participants with a total error rate of more than 10% were excluded. Incorrect responses (4.62% of total trials), as well as anticipatory responses and delays (0.5% of total trials, of which response times less than 100 or greater than 2,000 ms) were filtered prior to analysis.

**Fig. 2:**
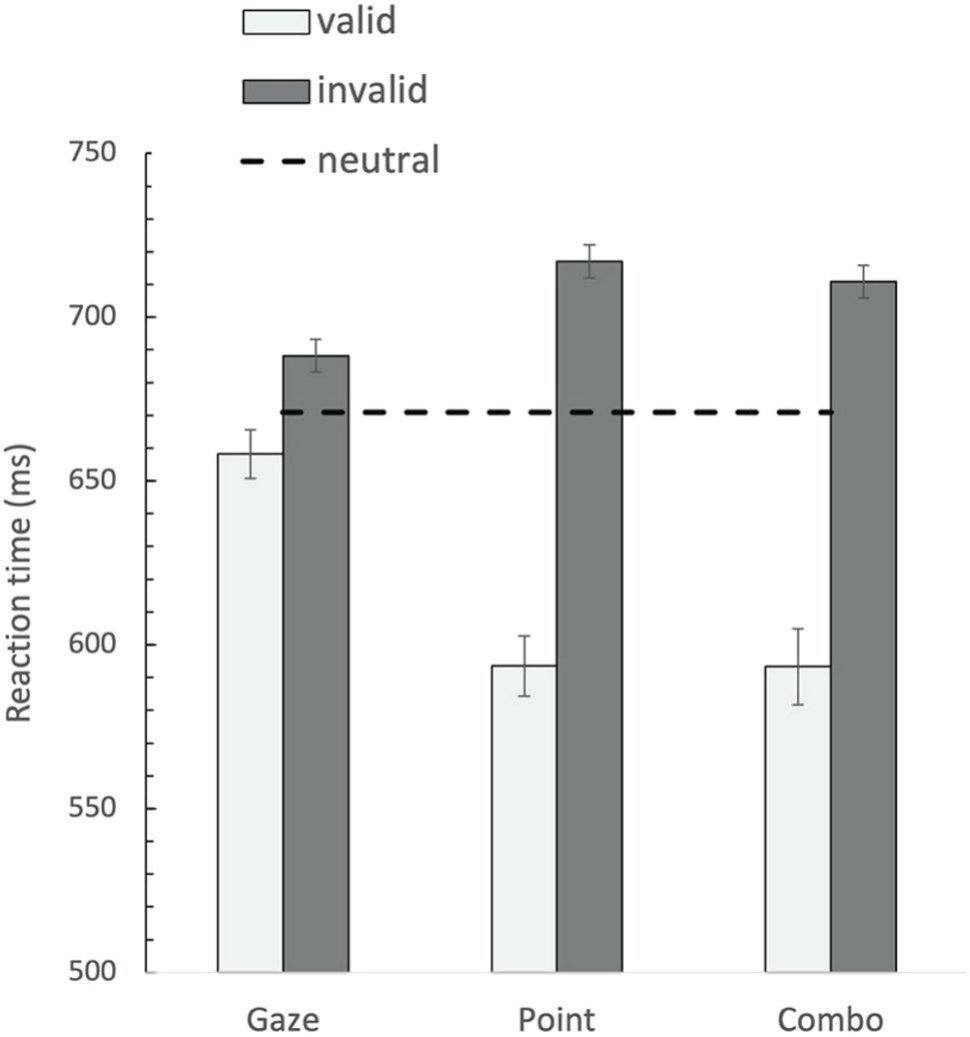
Mean response times for three cue-types and the neutral condition as a function of validity. Error bars represent within-subject error

Figure 2 shows the average response times (averaged across participants) as a function of the different experimental conditions. The RTs were subjected to a 3 (cue type: gaze, pointing, combo) × 2 (cue validity: valid, invalid) repeated-measures analysis of variance (ANOVA). The result showed the main effect of cue type, *F*(2, 54) = 4.85, *p* = .012, η^2^ = .152, and validity, *F*(1, 27) = 45.194, *p* < .001, η^2^ = .626, with faster responses on combo-cue- and pointing-cue- than on gaze-cue-oriented trials (RTs 651 ms, 653 ms vs. 673 ms, respectively) and a main effect of validity, showing valid cues were faster than invalidly cued trials (RTs 630 ms vs. 694 ms, respectively). Additionally, the Cue Type × Cue Validity interaction was also significant, *F*(2, 54) = 19.153, *p* < .001, η^2^ = .415. This resulted from the evidence that the cue effect (RTinvalid – RTvalid) of the combo cue and pointing cue were much larger than that of the gaze cue (117 ms, 123 ms vs. 30 ms). A direct comparison between the gaze cue and the combination cue, showed that the combination cue has a greater effect size than gaze cue, *t*(27) = 4.561, *p* <.001, while it had no distinct advantage over pointing cue *t*(27) = −0.455, *p* = .653. A paired-sample *t* test between valid and invalid gaze-cue trials showed that the gaze cue did produce a reliable cueing effect in this experiment, *t*(27) = −2.516, *p* = .018.

We analyzed the effect of the visual field on participants’ task performance. The result of 3 (cue type: gaze, pointing, combo) × 2 (visual field: upper, lower) × 2 (validity: valid, invalid) repeated-measures ANOVA revealed the main effect of the visual field, *F*(1, 27) = 13.969, *p* < .001, η^2^ = .34, with faster RTs when the target is located at upper visual field rather than the lower visual field (RTs 645 ms vs. 679 ms, respectively). However, none of the interactions with visual field, cue type and validity reached significance (all *p*s > .48), indicating that potential differences in cue susceptibility were not modulated by visual fields.

### Discussion

Experiment 1 had three main results. First, the results indicated that all cues impacted attentional performance: valid trials were responded to faster than invalid trials, in the gaze-, pointing-, and combo-cue conditions alike. Second, the validity effect for the combination cue was similar to the validity effect for the pointing cue alone. This suggests that contrary to our hypothesis, the combination cue in which two cues were presented, did not provide additional benefits to performance when compared with the single cue condition. Third, the results showed that the pointing cue was much more powerful than the gaze cue; the effect was almost four times as big. The results furthermore showed that while individuals were more responsive to stimuli appearing in the upper visual field, consistent with the idea of an upper visual field dominance in visual search (Previc, 1990; Thomas & Elias, 2011), there were no interactions between cue type and visual field. This suggests that participants’ visual field sensitivity was not differentially influenced by the type of cue presented.

The results from Experiment 1 are not in line with prior findings showing that combination of cues can have greater impact on orienting than the effects of cues in isolation (Bai et al., 2020; Langton & Bruce, 2000). However, there are obvious differences between the gaze- and pointing-cue conditions in the present study that could have influenced the present results. For example, in terms of perceptual features, the gaze cue is small and subtle compared with the pointing cue, which is larger, closer to the target, and has a distinct outline. Based on low-level features, the pointing cue is much more salient than the gaze-cue effect. Related to this, it may be the case that the cue benefit elicited by the pointing cue was at ceiling, such that the aligned gaze cue may not have been able to provide any additional advantages. Additionally, the relative spatial position in relation to the centre of the display of the two cues could have inadvertently affected their relative impact. One could argue that the pointing cue was presented more centrally compared with the gaze cue and therefore processed better or quicker than the gaze cue. Then again, Hermens et al. (2017) showed that regardless of the relative spatial position of the cues, the cueing effect of pointing cue was greater than that of gaze cue. In their experiment both cues were presented in the periphery.

Experiment 1 was suboptimal also regarding the manipulation of visual field. The spatial specificity of a pointing cue is more succinct than that of the gaze cue. In other words, it is easy to perceive the difference between the character pointing up or down, while in case of the gaze cue, this was much less obvious. The question then is whether eye direction can have such a precise effect on attentional deployment. While the present data do not directly speak to this issue, it seems plausible to assume that the gaze cue was much less informative regarding whether the target would appear in the upper or lower visual field compared with the pointing cue. This may have further decreased the relative impact of the gaze cue relative to the pointing cue.

Experiment 2 was conducted to address some of these limitations. Experiment 2 used only two locations (left and right). This experiment set out to test whether the gaze cue could compete with the strong pointing effect when it cued a different location (i.e., complete opposite location) compared with the pointing cue.

## Experiment 2

The aim of Experiment 2 was to determine how the compatibility (aligned vs. conflicted directions) of combined cues affects biases in attention. The extent to which one of the cues is prioritized, can be derived from situations where the direction of the cues is posed against the other cue. Finding that one of the two competing cues has a stronger impact on attention, would suggest this cue is prioritized over the other cue. If both cues are equally important, one would predict that observers would randomly follow the direction of one cue and the impact of the conflicted cues would equal each other out. Based on the results of Experiment 1, which revealed a strong dominance of the pointing cue, we hypothesized that the pointing cue would have a greater impact on performance in the conflicting cue conditions than the gaze cue.

In Experiment 2, the gaze and pointing cue were always present in the cartoon, but cues could either be directed in the same or opposite directions. Additionally, based on the observation that visual field did not interact with the cue effect, target and distractor were presented at two locations only—left and right relative to the centrally presented cues.

### Methods

#### Participants

Thirty-one subjects (23 male, mean age 27 years) were recruited in exchange for a monetary reward using the Prolific website (prolific.co). Each subject had normal or corrected-to-normal eyesight. Informed consent of each participant was obtained prior to the study; the study was approved by the university Ethical Committee (protocol ERN-19-0260) and was conducted in accordance with the principles expressed in the Declaration of Helsinki.

#### Stimuli

**Fig. 3:**
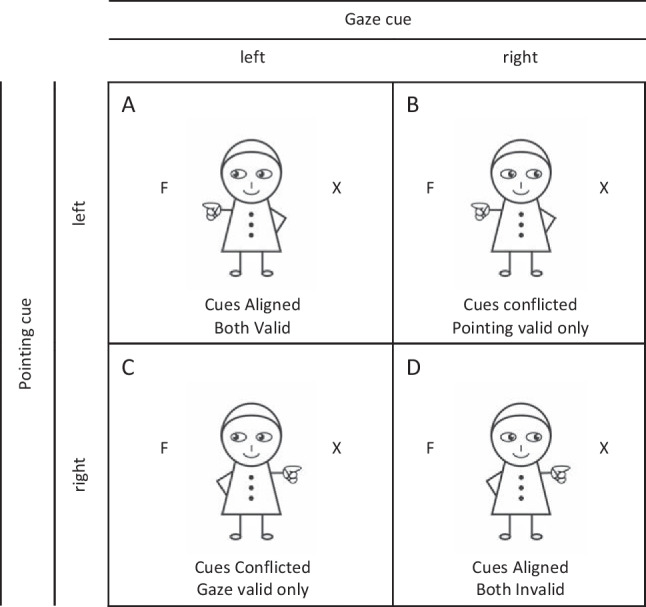
In addition to the neutral cue, there were four cueing conditions in Experiment 2. In Cues Aligned condition, gaze and pointing cues were directed towards same location, while in Cues Conflicted condition they were oriented in opposite directions. Depending on the target location, either both cues were valid (**A**) or invalid (**D**), or the Gaze cue was valid only (**C**), or the Pointing cue was valid only (**B**). Note, this figure shows only targets presented on the left side, but targets were presented equally often on the left and right side

Experiment 2 continued to employ the cartoon character from Experiment 1, but this time with three types of cue condition: neutral cues, two aligned cues, and two conflicting cues (Fig. 3). In the aligned cue condition, the gaze and pointing cues always pointed in the same direction. In 50% of all trials, the aligned cues were valid and in the 50% they were invalid, and thus unpredictable with respect to the upcoming target location. In contrast, in the conflicted cue condition, the two cues were oriented in opposite directions (e.g., gaze to the left and pointing hand to the right and vice versa). In the conflicted cue condition, the two cues had an equal chance of being valid or invalid. Either the gaze cue was valid and the pointing invalid, or the pointing cue valid and the gaze cue invalid. In other words, when the gaze cue was valid, the pointing cue was invalid, and vice versa. In addition to the four cue types, the neutral stimulus devoid of directional information, was presented to provide a baseline condition. The target letter (“F” or “H”) was assigned to one of two possible places (left or right) with equal probability. In the opposite location of the target, the distractor letter “X” appeared.

#### Design and procedure

Within-subject characteristics in Experiment 2 included cue compatibility (aligned or conflicted) and cue validity (valid, invalid). Specifically, there are four experimental conditions: both cues valid, both cues invalid, gaze cue valid only (i.e., pointing cue invalid), and pointing cue valid only (i.e., gaze cue invalid). Gaze and pointing cue were oriented in the same direction in half of the trials (aligned trials), while in the other half they were orientated in the opposite direction (conflicted trials). In the case of conflicting trials, the probability of gaze and pointing cues acting as a distractor was equal. In addition to the stimuli presenting directional information, neutral trials were presented containing no directional information (36 trials).

Experiment 2 followed a similar procedure as Experiment 1. A fixation point was presented for 500 ms, followed by the neutral image for 500 ms, followed by the critical cue image for 350 ms. After 350 milliseconds, the target letter (“F” or “H”) appeared in one of two possible locations to the left or right of the central cue. Each participant would initially complete 16 practical trials, followed by 144 experimental trials divided into nine blocks.

### Results

Three participants with excessive error rates (more than 10%) were excluded from the final analysis. Incorrect responses (4.17% of total trials), as well as anticipatory responses and delays (0.12% of total trials, whose response times less than 100 or greater than 2,000 ms) were filtered before the final analysis.

**Fig. 4:**
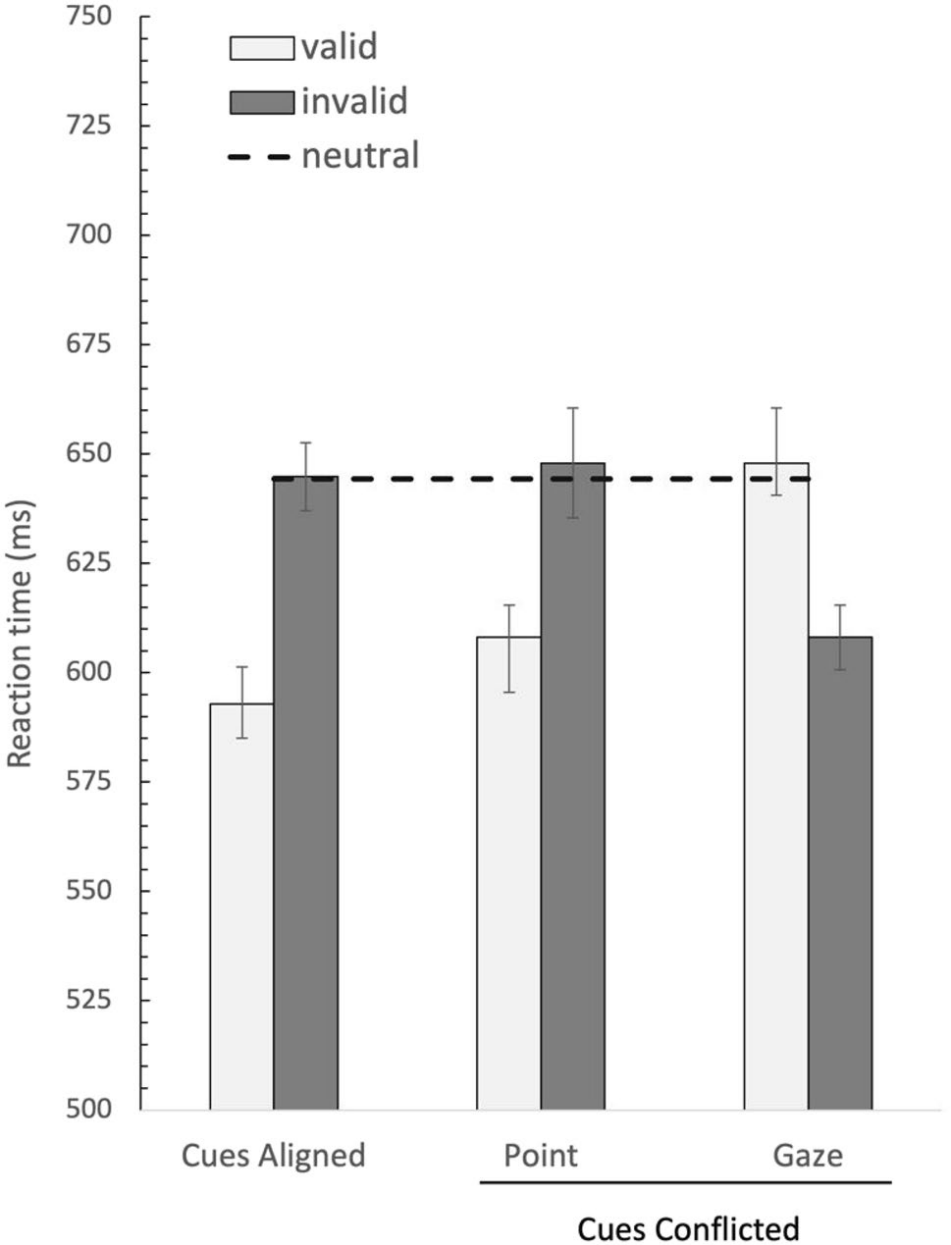
Average reaction times as a function of the different cue condition in Experiment 2. Note that in the Cues Conflicted condition, the gaze conditions mirror those of the point condition, as the valid pointing-cue condition means the gaze cue is invalid. Error bars represent within-subject error

The average response time as a function of the cue conditions and validity are presented in Fig. 4. To investigate the effect of cue compatibility, the mean RTs were subjected to a 2 (cue compatibility: aligned, conflicted) × 2 (validity: pointing-cue valid, pointing-cue invalid) repeated-measures ANOVA. Note that in the conflicting condition, the results were analyzed referenced to the pointing cue; in the conflicted pointing-cue valid condition, the gaze cue was invalid, while in the conflicted pointing-cue invalid condition, the gaze cue was valid. This resulted the main effect of validity, *F*(1, 27) = 16.359, *p* < .001, η^2^ = .377, with faster RTs on validly cued trials (both-cues valid & pointing-cue valid only) than invalidly cued trials (RTs 644 ms vs. 593 ms, respectively). There was no main effect of cue compatibility (*F* < 1). Additionally, the interaction between cue compatibility and validity did not reach significance, *F*(1, 27) = 1.36, *p* = .254.

A paired-samples *t* test yielded significance showed a reliable cueing effect for the pointing cue, showing that the valid pointing cue was responded to faster than the invalid pointing cue, *t*(27) = 2.716, *p* = .011, (RTs 608 ms vs. 647 ms, respectively). Comparing the performance against the neutral trials, a reliable difference was found between the valid pointing-cue condition and the neutral condition (608 ms vs. 644), *t*(27) = 2.427, *p* = .022. In contrast, there was no reliable difference in RTs between the neutral and invalid pointing cue (647 ms vs. 644 ms).

### Discussion

The results of Experiment 2 showed that when the two cues were directed to different locations, and the pointing cue was prioritized over the gaze cue. Compared with the condition where both cues were directed to the same location, the conflicting gaze cue had no impact on task performance, such that attention was biased in line with the pointing cue only. These results are in line with findings from Hermens et al. (2017) showing that an inconsistent gaze cue had no discernible effect on responses to the pointing cue; while, when participants were required to respond to the gaze cue, a conflicting pointing cue did significantly interfere with performance (Hermens et al., 2017).

Similar to the concerns raised in the discussion of Experiment 1, it may still be argued that the gaze cue in the present study is too subtle and much less salient compared with pointing cue, explaining the reduced impact of this cue when it competes with the pointing cue. To test this idea, Experiment 3 investigated the impact of a potentially more salient social cue—namely, head direction. In contrast to the gaze cue where direction involved no changes in outline and is expressed by minimal movement of the pupil in the eyes, direction in the head cue is indicated by a complete change in profile. This should be perceived as a greater change, be more salient and therefore predicted to have more impact. This idea is in line with Hermens et al. (2017) who have suggested that head cues, which incorporate all the prominent facial features concurrently, can be more potent than simple gaze cues. The aim of Experiment 3 is to test whether increased cue saliency subsequently increases competition for attentional resources with the pointing cues.

## Experiment 3

The head and pointing cues were always presented together, but cues could either be directed in the same or opposite directions. Compared with the gaze cue in Experiment 2, we predicted that the head cue would represent a more salient cue. The head cue was predicted to be more competitive in biasing spatial attention and result in a greater interference cost relative to the pointing cue.

### Participants

Thirty-three subjects (19 male, mean age 26 years) were recruited in exchange for a monetary reward through the Prolific website (prolific.co). Each subject had normal or corrected-to-normal eyesight. Informed consent of each participant was obtained prior to the study; the study was approved by the university Ethical Committee (protocol ERN-19-0260) and was conducted in accordance with the principles expressed in the Declaration of Helsinki.

### Stimuli

**Fig. 5:**
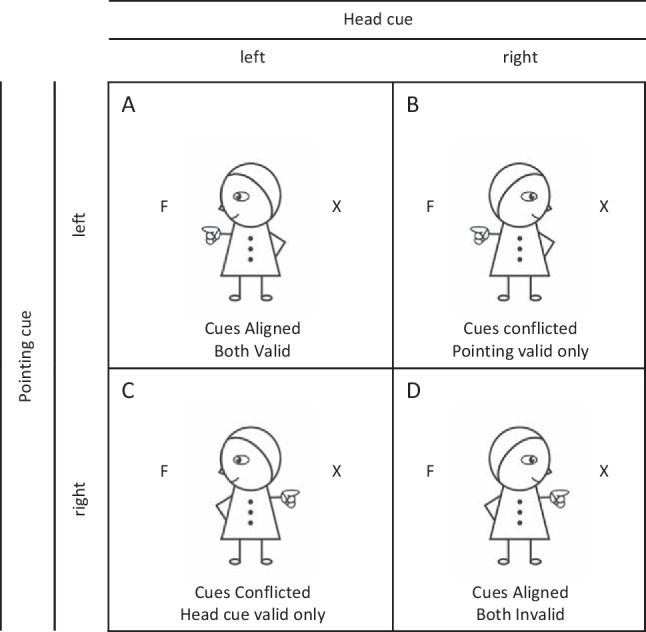
In addition to the neutral cue, there were four cueing conditions in Experiment 3. In Cues Aligned condition, gaze and head cues were directed towards same location, while in Cues Conflicted condition, they were oriented in opposite directions. Depending on the target location, either both cues were valid (**A**) or invalid (**D**), or the Gaze cue was valid only (**C**), or the Head cue was valid only (**B**). Note, this figure shows only Targets presented on the left, targets presented equally often on the left and right side

The head cue simulated a human head changing position horizontally 90 degrees to the left or right, with one eye visible only at the end of the “movement” (Fig. 5). The pointing cue was identical to that used in earlier experiments. Like Experiment 2, Experiment 3 involved a neutral cue in addition to the four experimental cueing conditions. In the aligned cue condition, the direction of the pointing and head cues were directed to the same location. In 50% of these trials, the aligned cue direction was valid and in the 50% of the trials it was invalid; cue direction was unpredictable with respect to the upcoming target location. In contrast, in the conflicted cue condition, the two cues were oriented in opposite directions (e.g., pointing to the left and head direction to the right). In the conflicted cue condition, the two cues had an equal chance of being valid or invalid. Either the head cue was valid and the pointing invalid, or the pointing cue valid and the head cue invalid. In other words when the head cue was valid, the pointing cue was invalid, and vice versa. A neutral stimulus devoid of directional information provided a baseline condition.

### Design and procedure

Within-subject factors in Experiment 3 included cue compatibility (aligned, conflicted) and cue validity (valid, invalid). Each cue’s likelihood of validity was the same as in Experiment 2. Furthermore, Experiment 3 followed the same procedure as Experiment 2. Each participant would initially complete 16 practical trials, followed by 144 experimental trials (36 neutral trials) divided into nine blocks.

### Results

Two participants with excessive error rates (more than 10%) were excluded. Incorrect responses (3.57% of total trials), as well as expectations and delays (0.1% of total trials, of which response times less than 100 or greater than 2,000 ms) were filtered out prior to analysis.

**Fig. 6:**
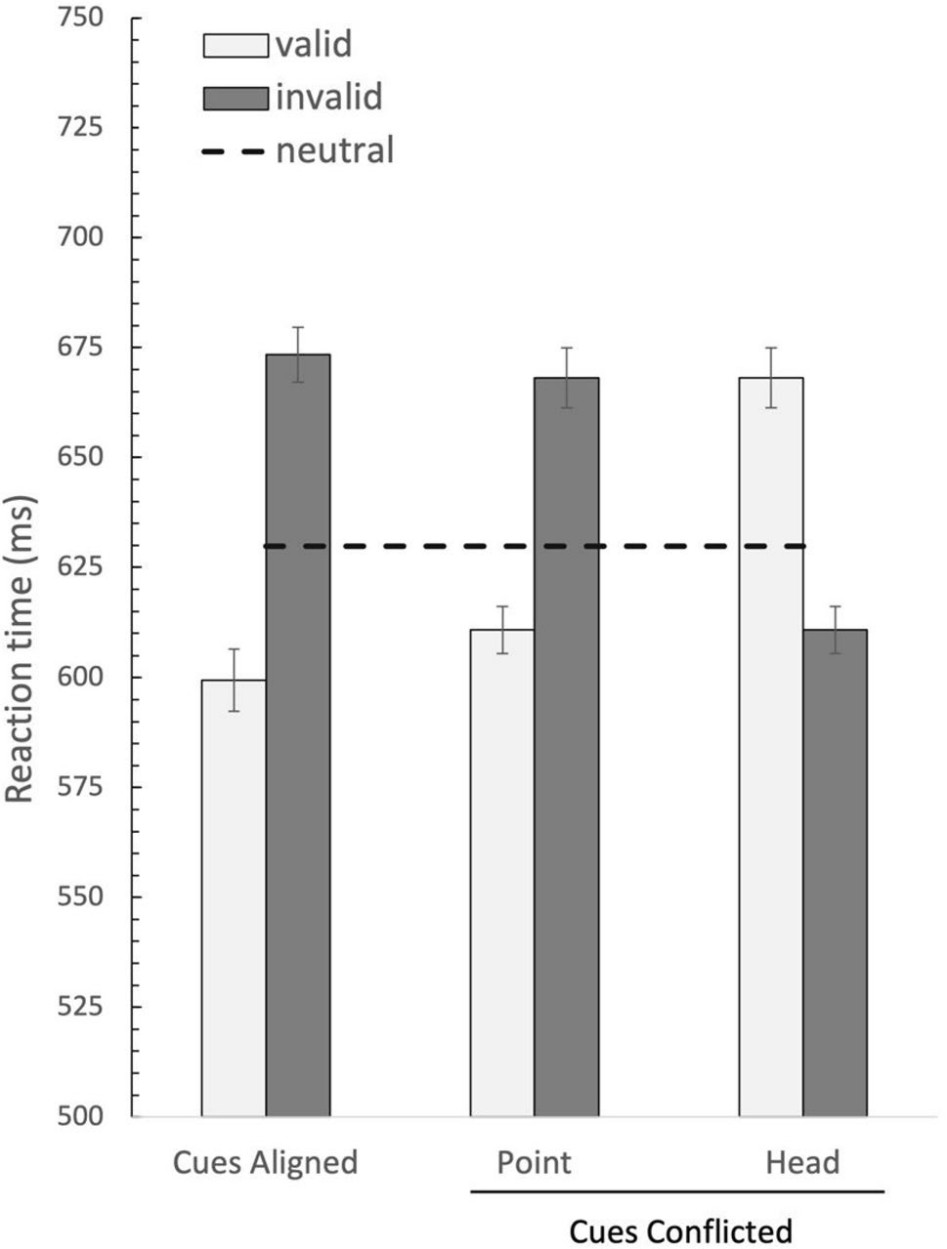
Average reaction times as a function of the different cue conditions in Experiment 3. Note that in the Cues Conflicted condition, the head conditions mirror those of the pointing condition, as the valid pointing condition means the pointing cue is invalid. Error bars represent within-subject error

The results of Experiment 3 are depicted in Fig. 6. The procedure of analysis was the same as in Experiment 2. First, the mean RTs were subjected to a 2 (cue compatibility: aligned, conflicted) × 2 (validity: pointing-cue valid, pointing-cue invalid) repeated-measures ANOVA. Note that in the conflicting condition, the results referenced to the pointing cue; in the conflicted pointing-cue valid condition, the head cue is invalid, while in the conflicted pointing-cue invalid condition, head cue is valid. The result demonstrated the main effect of validity, *F*(1, 30) = 49.92, *p* < .001, η^2^ = .625, with faster RTs on validly cued trials (both-cues valid & pointing-cue valid only) than invalidly cued trials (RTs 649 ms vs. 584 ms, respectively). There was no main effect of cue compatibility, *F* < 1. Additionally, the Cue Compatibility × Validity interaction was not significant, *F*(1, 30) = 1.94, *p* = .17.

RTs to the pointing-cue valid condition was tested directedly against the invalid pointing cue condition using the paired-samples *t* test. The results yielded a significant effect, *t*(30) = −5.943, *p* < .001, with faster RTs on valid pointing cue than invalid pointing cue (RTs 611 ms vs. 668 ms, respectively). Performance was also compared with the neutral trials by paired-sample *t* test. Under conflicted cueing conditions, the invalid pointing cue led to significantly slower responses than did the neutral cues (669 ms vs. 630 ms), *t*(30) = 3.095, *p* = .004, whereas there was no difference between valid pointing cue and the neutral condition (611 ms vs. 630 ms), *t*(30) = 1.62, *p* = .12.

Finally, based on the results of Experiments 2 and 3, we conducted a 2 (cue compatibility: aligned, conflicted) × 2 (validity: valid, invalid) × 2 (experiment: Exp2, Exp3) repeated-measures ANOVA to explore the consistency of the pointing effect over the two experiments. This analysis yielded a main effect of validity only, *F*(1, 57) = 58.73, *p* < .001, η^2^ = .507. There was no main effect of Experiment (*F* < 1), no interaction between cue compatibility and experiment (*F* < 1), nor between validity and experiment, *F*(1, 57) = 1.84, *p* = .18. The interaction between these three factors failed to reach significance (*F* < 1). A direct comparison between the cue effect (RT_invalid_ − RT_valid_) in the conflicting gaze versus head cue condition was not reliable, (45 vs. 55 ms), *t*(57) = 1.01, *p* = .32. This indicated no significant difference in the overall pattern of data between Experiment 2 and Experiment 3.

### Discussion

In Experiment 3 the head cue was tested against the pointing cue. Relative the Experiment 2 which tested the gaze cue, it was hypothesized that the head cue would be more salient and therefore more competitive in biasing spatial attention. While the head cue involved a more dramatic change in visual stimulation, the results of Experiment 3 produced very similar results to Experiment 2. Comparable with the gaze cue, the head cue had a negligeable effect on performance, which was dominated by the pointing cue and was completely independent of the validity of the pointing-cue.

**Fig. 7:**
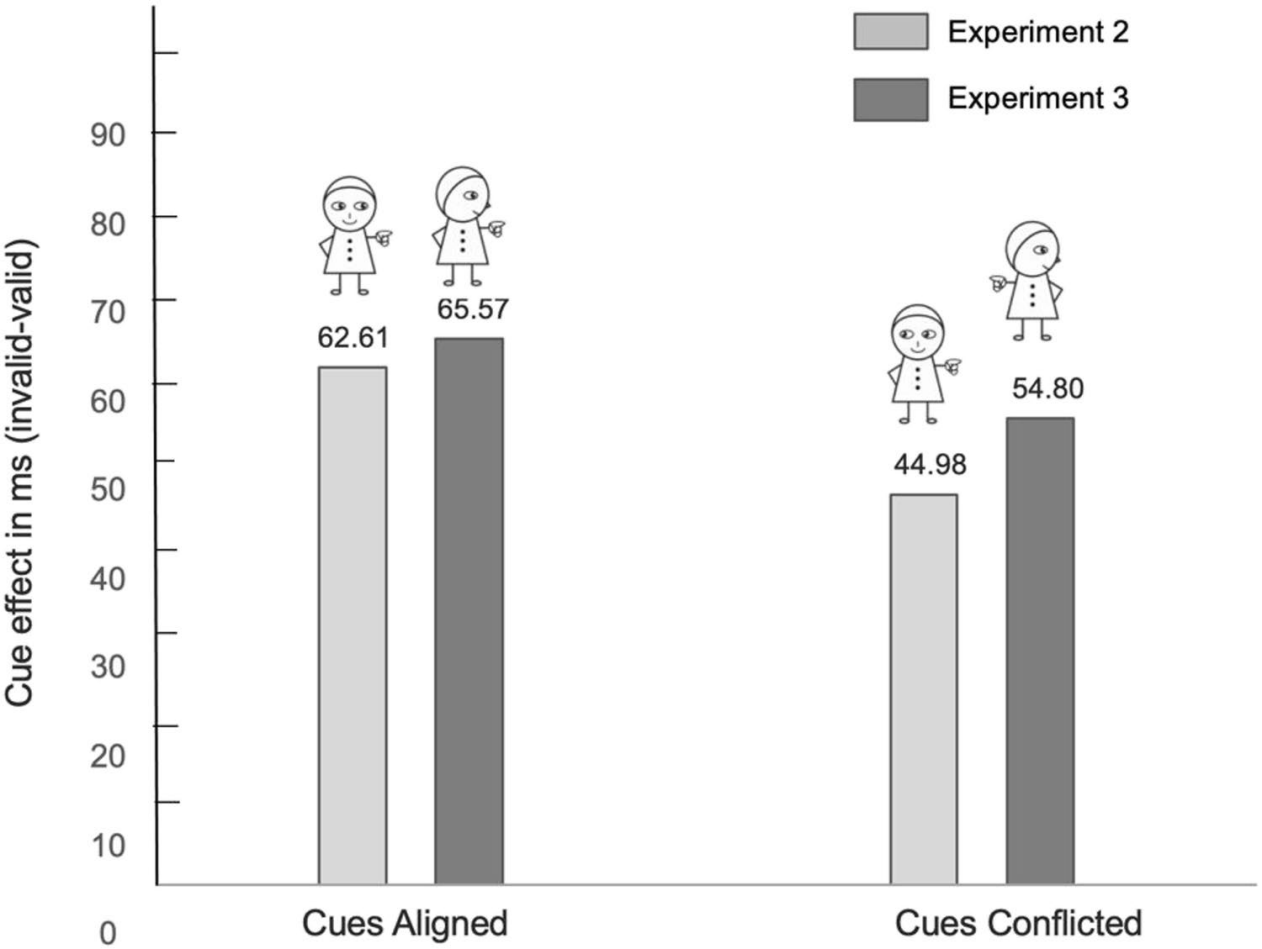
The cue effect in ms (RT_invalid_ – RT_valid_) of the Cues Aligned and Cue Conflicted conditions in both Experiment 2 and Experiment 3. In the Cue Conflicted condition, the results are referenced to the pointing-cue condition. In Experiment 2, the pointing cue was tested against the gaze cue. In Experiment 3, the pointing cue was tested against the head cue

Comparing Experiment 2 with Experiment 3, while there was a numerical difference in the validity effect for the impact of the pointing cue (see Fig. 7), suggesting that the incongruent pointing cue might lead to increased interference compared with the incongruent gaze cue, this was not statistically reliable. While we do not have a direct comparison between the cue effect of the gaze and pointing cues in isolation, these results suggest that the impact of the head-direction cue is very similar in strength as the gaze cue. One thing to note, in Experiment 2, when the pointing cue was tested against the gaze cue, we observed a benefit for the valid pointing cue relative to neutral condition. In Experiment 3, when the pointing cue was tested against the pointing cue, evidence pointed toward a reliable cost in the invalid pointing cue condition relative to the neutral condition. This may indirectly suggest that the invalid head cue has a stronger impact on attention compared with the gaze cue, as it is able to draw attention away relative to the neutral condition. The invalid gaze cue does not have this impact on attentional deployment, showing no additional costs when it is invalid relative to the neutral condition.

The results of Experiment 3 are not in line with evidence from Hermens et al. (2017), who concluded the pointing cue is a more robust version of the gaze cue, due to its distinct outline compared with gaze cue. Our results are in consistent with a video-based cueing study conducted by Ouwehand et al. (2015), who showed priority over the processing of pointing gestures compared with head direction. In this work, a human model was programmed to either rotate her head or position her finger towards a specific area of a screen. The results showed that participants’ attention to task-relevant areas increased when the model provided pointing gestures but showed a reduced impact in response to head rotation in isolation. Like the present study, this work demonstrated that pointing cues are more effective in directing attention than pointing cues. This is also in line with a study that investigated learning in video lectures, showing that pointing gestures improve learning regardless of the directed gaze or head directions (Pi et al., 2019).

In sum, integrating the findings from Experiments 2 and 3, we concluded that the validity of the pointing cue had a dominant effect on participants’ task performance, regardless of the presence of other conflicting social cues.

## General discussion

The primary purpose of the present study was to examine the interaction between different social cues. Evidence from three experiments revealed a dominance of the pointing cue over the gaze cue and pointing cue in the present stimuli set. Experiment 1 revealed that the pointing cue alone revealed a cueing effect that was equal in size regardless of whether the location was additionally cued by gaze. In Experiment 2, when the gaze cue and pointing cue were posed directly against one another, the results showed that the pointing cue determined task performance regardless of whether the direction of the gaze cue was aligned or conflicting. A similar result was found in Experiment 3, where instead of the gaze cue, a pointing cue was used. Even though the pointing cue was more dynamic in terms of low-level features, it had a negligeable effect of performance. Again, the pointing cue was driving the overall validity effect.

Several studies have similarly demonstrated an imbalance in the impact of nonpredictive gaze cues and head cues on attentional biases (Hermens et al., 2017; Langton & Bruce, 2000; Nummenmaa & Hietanen, 2009). The present study showed that the directing roles of the eyes and head were overshadowed by pointing gestures. Specifically, the gaze and pointing cues did not compete with selection, and participants processed the pointing cue preferentially. Differences between the present results and previous work might have occurred because of methodological differences (e.g., Hermens et al., 2017; Sato et al., 2010). In most of the previous work, participants were required to respond directly to one of the two cues that appeared in pairs, while a nontarget cue was designated as distractor or interfering stimulus. The impact of cues on response selection may be different than the impact of the cues in directing spatial attention. The main task in the present study was to respond to the identity of a letter presented in the periphery, and to ignore all social cues because they were task irrelevant. In contrast to previous work where one of cues was task irrelevant, the present study tested the impact of combination of cues on that were both unrelated to the main task.

There are many differences, perceptual and otherwise between the cues that can also help to explain the present findings. First, it may be that the pointing cue is more salient than either the gaze- or head-direction cue. Hermens et al. (2017) have suggested the unique outline might explain the potent role of pointing gestures. In their work, conflicting conditions involved the face, eyes, hand, and head direction; these were presented pairs as separated stimuli above or below fixation. They concluded that the cue with a distinct outline (e.g., pointing hand cue) has the strongest inference on the deployment of attention. Second, in addition to the external outline, the motion of the pointing hands may be more prominent compared with movements of the eyes and head (Gregory & Hodgson, 2012). Third, the pointing cue may have a more spatially specific impact on spatial attention, compared with the gaze and pointing cue, and be able to cue a more precise or confined area in space. Fourth, pointing with the index finger to express spatial information is a cross-culturally gesture that is reinforced daily, whereas eye gaze and head motions may be considered relatively more complicated stimuli which may require more time to comprehend (Fan et al., 2018).

Another reason that could explain why observers seem to place more weight to the pointing cue is based on implied intentionality. Eye movements are cheap, and changes in head-direction and orientation are similarly relatively inexpensive movements that may be elicited without awareness. In contrast, pointing movements using the arm and index finger typically are fully intentional, elicited primarily with purpose or strategy. The idea here is that the pointing movements elicits a greater cue effect because they are regarded as more purposeful by the observer. One way to investigate this idea may be to manipulate the proportion with which cues appear and the degree to which they are valid (e.g., see Nummenmaa & Hietanen, 2009). By manipulating the validity of the cues independently, it may be possible to equalize the strength of the pointing cue to the gaze cue, yielding an indirect estimate of how much stronger the pointing cue is in certain situations. Another way to investigate the impact of the contribution of low-level features is to tease apart the role of low-level saliency and intentionality in the pointing cue. For example, one could compare a situation where the low-level stimulation of a lateralized arm is comparable to that of the pointing cue but does carry the same directional meaning because it is holding a cup or a flower. The idea being that an outstretched arm holding an object bears no explicit directional information. Alternatively, the present stimuli can be modified to create a situation where the pointing does not involve a distinct outline but is presented centrally in front of the body. We are currently testing these manipulations to investigate the relative role of low-level features and saliency in biases of social cues (Higgins & van Zoest, 2023). Results may further depend on which response is required from the participants. For example, Crostella et al. (2009) found a relation between the cueing effect and the type of response—gaze stimuli interfered with oculomotor performance, while hand stimuli interfered with pointing performance. The prediction is that when the dependent measure is landing position of eye movements instead of manual RT, the gaze-cue effect may be larger compared with the pointing cue (e.g., Bonmassar et al., 2019).

One limitation of the stimulus set used in the present study is that the cartoon has low ecological validity, which makes it difficult to generalize to real-world situations. At the same time, other research has suggested that performance in gaze cuing is very much comparable regardless of whether real-life photos are used or schematic stimuli (Sato et al., 2007). Even though the stimuli are animated, a benefit of the present stimuli set is that they are child friendly. Having established the results in young adults, this work can probe further developmental research on the combination of social cues in typically developing children as well as children with atypical or neurodiverse backgrounds, such as those with autism, for example (e.g., Bayliss & Tipper, 2005; Corkum & Moore, 1995; Daum et al., 2013; Farroni et al., 2003; Gregory et al., 2016; Morgan et al., 2021; Ristic et al., 2005; see also Nation & Penny, 2008). In the case of deaf individuals, evidence suggests that susceptibility to social cues might change throughout development. Whereas young deaf children are found to be more sensitive to the gaze cue compared with their hearing peers (Pavani et al., 2019), deaf adults have been reported to be less susceptible to the gaze cue compared with their hearing peers (Heimler et al., 2015; however, see Bonmassar et al., 2021). The question is whether these developmental differences are similar in response to pointing movements in the deaf. Modulated by sign-language experience, deaf observers may perceive the pointing cue as very different compared with the gaze cue. Studying the combination of cues together provides insight in the potential interaction between different cues, something that may be altered by atypical sensory experience and environmental learning (Pavani et al., 2019).

In conclusion, while all tested social cues biased spatial attention, the present results suggests that the pointing cue is most powerful in prioritizing attention. This work presents a versatile new way to study the impact of the combination of social cues, which can probe further research in developmental psychology of social attention, as well as new avenues for research in populations for whom social attention may be atypical.

## Supplementary information

ESM 1 (DOCX 12 kb)

## Data Availability

Stimuli materials and data are available on https://osf.io/8tkfx/
