# Supplementary material for: Combining social cues in attention: Looking at gaze, head, and pointing cues
Source: Atten Percept Psychophys. 2023 Feb 27;85(4):1021–33. doi: 10.3758/s13414-023-02669-6 (PMC10167180; doi:10.3758/s13414-023-02669-6)
Supplement: Supplementary file 1 — (DOCX 12 kb) [file 13414_2023_2669_MOESM1_ESM.docx]

Supplementary material

Stimuli materials and data are available online (https://osf.io/8tkfx/).
